# Macrofungi of Mata da Margaraça (Portugal), a relic from the Tertiary Age

**DOI:** 10.3897/BDJ.7.e38177

**Published:** 2019-10-03

**Authors:** Bruno Alexandre Fragoso Natário, Rogério Louro, Celeste Santos-Silva

**Affiliations:** 1 Biology Department, Macromycology Laboratory, Instituto de Ciências Agrárias e Ambientais Mediterrânicas, University of Évora, 7002-554, Évora, Portugal Biology Department, Macromycology Laboratory, Instituto de Ciências Agrárias e Ambientais Mediterrânicas, University of Évora, 7002-554 Évora Portugal

**Keywords:** Ascomycota, Basidiomycota, Beira Litoral, Portugal, Laurisilva, post-fire

## Abstract

Mata Nacional da Margaraça represents a rare example where the Atlantic climate influences the perpetuity of a small enclave of a previously widespread laurel forest. The higher relative humidity values (> 80%), which are almost constant all year long and the mild temperatures, rarely exceeding 30°C, even in the dry season (June to September), create an insular-like effect. The biological communities of Margaraça exhibit a transitory character. The forest is dominated by *Quercus robur* and *Castanea sativa*, yet *Quercus suber*, although less frequent, can also be found. The laurel species, such as *Viburnum tinus*, *Ilex aquifolium*, *Laurus nobilis* and the Portuguese endemic Prunus lusitanica ssp. lusitanica, relics from the ancient Portuguese Tertiary, comprise the understorey. The present work represents, to the best of our knowledge, the first regional macrofungal species list of the Mata da Margaraça published to date. The recent fires that occurred in the area have provided the opportunity to study the post-fire communities. The surveys were carried out in 2004 and later in 2018-2019. A total of 271 species were registered as belonging to Basidiomycota (≈ 80%) and Ascomycota (≈ 20%). The most represented Basidiomycota families were Russulaceae, Mycenaceae and Agaricaceae and the most represented Ascomycota families were Pyronemataceae and Pezizaceae. The new records to Portugal add up to a total of 88 species and another 116 species are new records to the province of Beira Litoral. Post-fire fungi account for 17 of the total of 271 species registered in these studies and most of these species are new to Portugal.

## Introduction

Continental Portugal’s unique location – eastern border of the Atlantic Ocean – allows for the co-existence of two biogeographic regions (the Eurosiberian region and the Mediterranean region) with distinct bioclimatic features (Costa et al. 1998). Besides the predominant Mediterranean climatic influence in the country, there are, nevertheless, rare locations where the Atlantic influence supports the establishment and continuity of different plant communities, some of which are relics of ancient forests, dating back to the Miocene and Pliocene periods. In that context, the Mata Nacional da Margaraça (M.M.) represents a rare example where the Atlantic climate influences the perpetuity of a small enclave of a previously widespread laurel forest called Laurisilva within the landscape of the Serra do Açor, which largely has a Mediterranean climate. Hence, in 1982, the Serra do Açor became a protected landscape area (A.P.P.S.A.) to protect the M.M. Nowadays, the M.M. is included in the European Network of Biogenetic Reserves and is a Site of Community Importance (SCI) under the Nature 2000 network (PTCON00051). With a total area of 68 ha, the M.M. is a unique reserve of biodiversity and an important conservation site in Central Portugal.

M.M. is situated in the Iberian fold belt (Lourenço 1996), between two large faults and several small ones and it is mainly occupied by acidic soils, originating from granite and schist rocks. The M.M. is located between 450 and 800 m above sea level and has an N-NW orientation. These topographic, geomorphologic and geographic characteristics are unique compared to other mountain regions with Atlantic climate influence in North and Central Portugal. For instance, the higher relative humidity values (> 80%), which are almost constant all year long and the mild temperatures, rarely exceeding 30°C, even in the dry season (June to September), create an insular-like effect, that is translated into the isolation of the ecosystems and species of the M.M. from the surrounding areas of the Serra do Açor (Neves 2005).

The biological communities of M.M. exhibit a transitory character, with a diverse flora comprised of Atlantic, Eurosiberian and Mediterranean species. However, given its dominance, the *Rusco aculeati-Quercetum roboris* association, sub-association *Viburnetosum tini*, class *Querco-Fagetea*, order *Quercetalia roboris* and alliance *Quercion robori-pyrenaicae*, best describe the flora of the M.M. (Alves et al. 1998). As such, in its majority, the forest is dominated by the Atlantic and Eurosiberian arboreal species. *Quercus robur* L. and *Castanea sativa* Mill. occupy most of the inner part of M.M., yet *Quercus suber* L., although less frequent, can be found on the outskirts of the M.M. The understorey is comprised mainly of laurel species (e.g. *Viburnum tinus* L., *Ilex aquifolium* L., *Laurus nobilis* L. and the Portuguese endemic Prunus lusitanica L. ssp. lusitanica) which are relics from the ancient Portuguese Tertiary Laurisilva forests (Habitats Directive Annex B-I, 5230). Furthermore, Prunus lusitanica ssp. lusitanica, contained in the Red List of Vascular Flora of Portugal, has, in the M.M., the largest number of individuals within its distribution range. Other shrub species, such as *Cytisus* spp., *Erica* spp., *Calluna vulgaris* (L.) Hull, *Ulmus minor* Mill., *Prunus cerasus* L., *Prunus avium* L., C*orylus avellana* L. and *Arbutus unedo* L., are also present in the understorey of these woods. In M.M., some of them can reach far greater sizes than in the rest of their distribution range. The M.M. herbaceous stratum accounts for many of the endemic species and the majority is listed in the Habitats Directive (H.D.) for this SCI, such as *Eryngium duriaei* Gay ex Boiss, Lavandula stoechas L. ssp. luisieri (Rozeira) Rozeira, *Antirrhinum meonanthum* Hoffmans. & Link, *Veronica micrantha* Hoffmans. & Link (in H.D. Annex II) and *Murbeckiella sousae* Rothm. (in H.D. Annex IV). The Bryoflora on the site is particularly well represented, with more than 150 species described. Some of these species, like *Cephaloziella elegans* (Heeg) Schiffn., *Campylopus pyriformis* Brid., *Hypnum revolutum* (Mitt.) Lindb., *Plagiothecium nemorale* (Mitt.) A. Jaeger and *Plagiothecium succulentum* (Wilson) Lindb., are restricted to M.M. in the Portuguese territory. Moreover, the reduced distribution of the above-mentioned *Plagiothecium* species in the national territory highlights the importance of M.M. as a preclimatic forest system.

Until the 1960s, the A.P.P.S.A. suffered profound human-induced landscape changes: 1) Massive deforestation was undertaken in order to create pastures, agricultural lands and for edification; 2) Replacement of the main forest with *Pinus pinaster* Ainton. monocultures. However, the M.M. area was preserved in order to produce wood (*Castanea sativa* coppice stands) and to harvest the forest surplus production. Later, rural desertification led to a decrease in agricultural land and pastures, which greatly contributed to shrub encroachment and forest regeneration (Moreira et al. 2011), by favouring the ecological succession and promoting the natural values of A.P.P.S.A. and particularly of M.M. However, the cumulative organic matter deposited on the forest floor increased the risk of high-intensity fires (Pausas et al. 2008) and culminated in the forest fires of 1987 and 2017 which burned 90% of the M.M. area and threatened its biodiversity, particularly in the upper limit of the M.M. and in its outskirts where non-native species (*Pinus* spp. and *Acacia* spp.) are more frequent. The fire had almost no effect in the lower limit of M.M. where the main forest remained untouched and the M.M. recover capability was preserved.

To the best of our knowledge, the present work represents the first regional macrofungal species list of M.M. published so far and one of the few available for Portugal (e.g. Louro et al. 2009). Due to its singularity, M.M. is a remarkable location for examining and documenting the macrofungal communities, as a unique relic of the ancient forests that previously occupied most of the national territory. Additionally, the recent fires that occurred in the area have provided the opportunity to observe the post-fire communities which, before the fire (undisturbed period), were less likely to be detected. The addition of these species to the list is particularly important since little is known about post-fire macrofungi communities in Laurel forests.

## Material and methods

**Figure 1.**
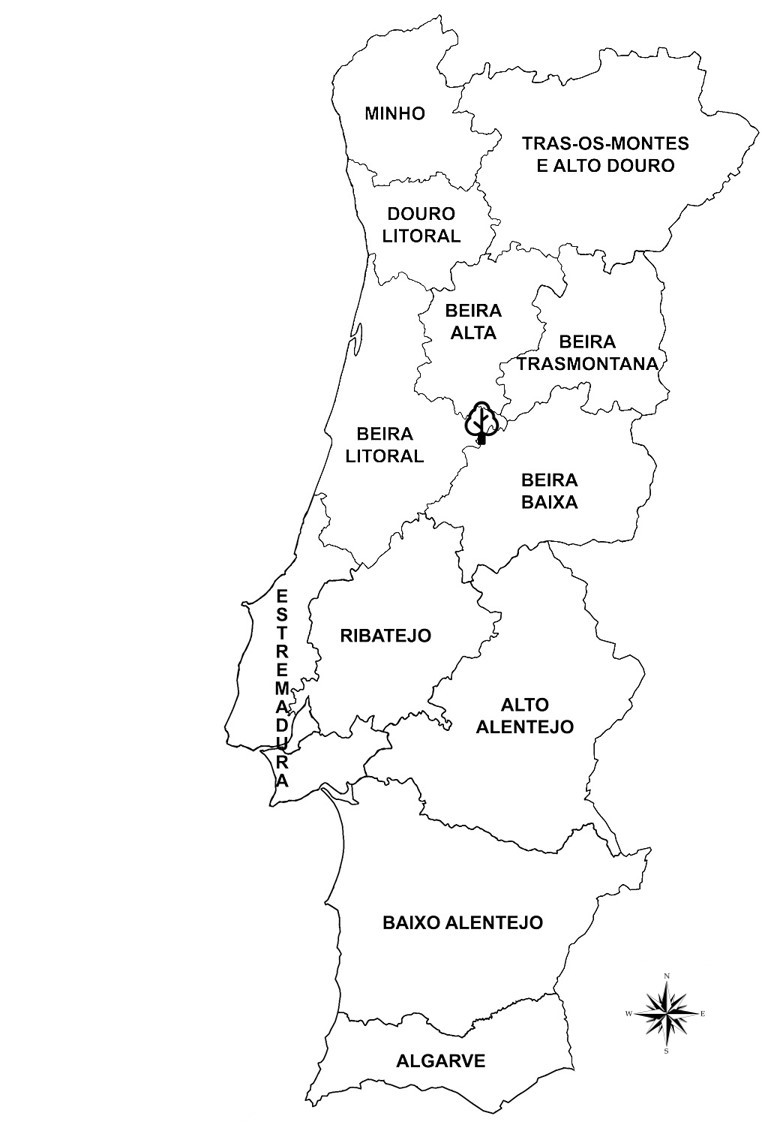
Map of the Portuguese territory. Map of the Portuguese territory divided by province. Mata da Margaraça located as a tree symbol.

The survey was conducted in the Mata Nacional da Margaraça (40°12.9781’ N, 7°55.1349’ W) (Fig. 1), between May 2018 and May 2019, with sampling occurring in the most favourable months, Spring (March-May) and Autumn (October-December). After the most recent wildfire (October 2017), 75 × 25 m^2^ plots were randomly distributed, within a fire severity range of values (Botella-Martínez and Fernández-Manso 2017). From the 75 preliminary plots, only 66 were surveyed due to topographic constraints. All the specimens within the plot area were harvested, stored under 4ºC and processed within twenty-four hours. These specimens were then preserved and deposited in Évora University herbarium (UEVH- FUNGI). Macrofungi nomenclature follows the Catalogue of Life (2019) and Kirk (2019). Current species distribution areas were consulted in Calonge (1998), Centro de Micologia da Universidade de Lisboa (2002), Global Biodiversity Information Facility Data Portal (2019) and other local publications and follows Castroviejo et al. (1986) usage of Iberian territory division into provinces.

Complementarily, a preliminary unpublished study, conducted by the Instituto para a Conservação da Natureza e das Florestas, was consulted. The study occurred in 2004, fortnightly, except for the months of July and August, in 10 × 100 m transects scattered throughout the 3 different biotopes, dominated respectively by *Quercus robur*, *Castanea sativa* and Prunus lusitanica ssp. lusitanica. All the specimens were collected and deposited in an exsiccatae personal herbarium (Gama 2004).

The species are arranged alphabetically, according to the higher taxonomic placement Phylum, Order, Family. For each species, the respective trophic group, putative host species (for mycorrhizal and parasitic species) and deposit number of UEVH- FUNGI were assigned. The species, referred to as novelties, were divided into species new to Portugal and new to the Beira Litoral province (B.L.) (Fig. 1). The post-fire species were identified according to Dix and Webster (1995), Sumorok (2001), Robinson et al. (2008) and Claridge et al. (2009) (Suppl. material 1).

## Results and Discussion

**Figure 2.**
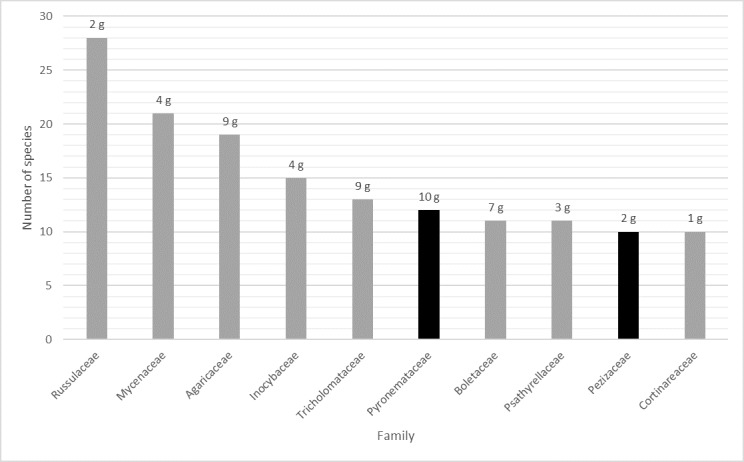
The ten most speciose families. Number of species of the ten most numerous families and number of genera (g) from each family. Grey bars refer to Basidiomycota families and black bars refer to Ascomycota families.

A total of 272 macrofungal species, representing 127 genera and 59 families, were recorded during these two studies, belonging to Basidiomycota (≈ 80%) and Ascomycota (≈ 20%) (Suppl. material 1). Considering the trophic groups, saprophytic species slightly outnumber the mycorrhizal species (1.36:1). The most represented Basidiomycota families were Russulaceae, Mycenaceae and Agaricaceae, accounting for 30% of all Basidiomycota species (Fig. 2), all well speciose families. *Russula* and *Lactarius* are mycorrhizal genera, well represented in Fagaceae dominated forest (Sarnari 2007). Mycenaceae and Agaricaceae comprise many species growing on decaying hard wood, such as *Quercus* spp. and *Castanea sativa* or associated with moss (Aronsen and Læssøe 2016). The most numbered Ascomycota families were Pyronemataceae and Pezizaceae, accounting for ≈ 50% of all Ascomycota species (Fig. 2). These species are greatly associated with forest ecosystems and some of them are post-fire species, such as *Anthracobia macrocystis* (Cooke) Boud., *Peziza praetervisa* Bres. and *Pyronema omphalodes* (Bull.) Fuckel.

Parasitic species were the least represented trophic group, with only six species, *Desarmillaria tabescens* (Scop.) R.A. Koch & Aime, *Rhizina undulata* Fr., *Thyronectria aquifolii* (Fr.) Jaklitsch, *Cordyceps militaris* (L.) Fr., *Phaeotremella foliacea* (Pers.) Wedin J.C. Zamora & Millanes and *Tremella mesenterica* Retz. Except for *C. militaris* (insect parasite) and *P. foliacea* and *T. mesenterica* (both fungal parasites), the other species are plant parasites. These species occurred frequently during sampling periods, except for Tremellaceae species.

The new records for Portugal add up to a total of 74 species and another 116 species are new records to B.L. (Suppl. material 1). The phylum Ascomycota is the least studied worldwide and even less so in Portugal. This fact is reflected in the number of novel Ascomycota species described in this study in which 44 are new records to B.L. and 22 of these are new records to Portugal. Due to the inconspicuous nature of most of these species (small dimensions and ephemerality of the carpophores) (e.g. *Bisporella citrina* (Batsch) Korf & S.E. Carp., *Lanzia echinophila* (Bull.) Korf and *Rutstroemia firma* (Pers.) P. Karst.), they are often overlooked and uncommon in species lists. The *Morchella* genus, in contrast, is well studied in most countries but represents an important novelty to B.L. Post-fire conditions were important for the development of some species (Dix and Webster 1995, Sumorok 2001, Robinson et al. 2008 and Claridge et al. 2009), such as *Anthracobia macrocystis* (Cooke) Boud., *Ascobolus carbonarius* P. Karst., *Morchella* spp., *Peziza* spp., *Plicaria endocarpoides* (Berk.) Rifai, most of which are novelties to Portugal.

Fifty-two novel Basidiomycota species were registered to Portugal and 94 to B.L. *Leucoagaricus crystallifer* Vellinga is especially worthy of mention since it is an extremely uncommon species in Europe, yet its rare observations are documented all over Europe (Vellinga 2000). In addition to *Leucoagaricus crystallifer*, species like *Cortinarius balteatocumatilis* Rob. Henry ex P.D. Orton, *Conocybe vestita* (Fr.) Kühner, *Cortinarius caperatus* (Pers.) Fr., *Crepidotus autochthonus* J.E. Lange, *Marasmius cohaerens* (Pers.) Cooke & Quél., *Marasmius epiphylloides* (Rea) Sacc. & Trotte, *Mycena pearsoniana* Dennis ex Singer, *Mycena pseudocorticola* Kühner, *Typhula quisquiliaris* (Fr.) Henn., *Pseudocraterellus subundulatus* (Peck) D.A. Reid and *Hymenochaetopsis tabacina* (Sowerby) S.H. He & Jiao Yang are representative of M.M. singular features. These species are associated with more humid and less variable climates, like the conditions found in M.M., which are uncommon in the rest of the country. Other species, such as *Boletus reticulatus* Schaeff., *Bovista plumbea* Pers., *Clitocybe costata* Kühner & Romagn., *Coprinellus domesticus* (Bolton) Vilgalys, Hopple & Jacq. Johnson, *Cortinarius trivialis* J.E. Lange, *Hygrocybe russocoriacea* (Berk. & T.K. Mill.) P.D. Orton & Watling, *Lycoperdon excipuliforme* (Scop.) Pers., *Mycena abramsii* (Murrill) Murrill, *Russula cyanoxantha* (Schaeff.) Fr., *Tremella mesenterica* Retz. and *Xylaria hypoxylon* (L.) Grev., are novelties to B.L. and reflect the lack of macrofungi studies in this area. These species are very common in the rest of the country but have not been documented for B.L. until now.

The post-fire fungi account for 17 of the total 271 species registered in these studies (e.g. *Ascobolus carbonarius* P. Karst., *Morchella elata* Fr., *Peziza praetervisa* Bres., *Pholiota brunnescens* A.H. Sm. & Hesler.) (Suppl. material 1). The addition of these species was an important enrichment to this checklist, since 7 of them represent novelties to Portugal (e.g. *Morchella eximia* Boud., *Pholiota brunnescens*, *Plicaria endocarpoides* (Berk.) Rifai) and were unlikely to be recorded otherwise.

## Conclusions

FUNGI comprise one of the most megadiverse biological groups and one of the most understudied and misunderstood. In a period where biodiversity loss is one of the main environmental challenges facing the planet, halting the loss of biodiversity and the degradation of ecosystem services by 2020 seems to be, nowadays, a more and more impossible task. The panorama seems even grimmer as climate change is deeply altering the geographical redistribution of plant and animal species and causing extinctions in the profoundly fragmented world of today. Under these premises, undertaking biological surveys that summarise the latest information on the status and trends of biodiversity, especially in areas of high biodiversity value in Natura 2000 sites and encouraging the publication of regional checklists on such high nature value areas, is of the utmost importance for the maintenance and preservation of biodiversity. In that context, the present work represents a contribution to the mycological knowledge of one of the most singular protected areas in Portugal, that harbours almost three hundred macrofungal species in a relatively small area. The wildfires that occurred in this area highly threatened the mycological diversity and subsequently their host species (Natario et al. unpublished data), most of which are obligatory mycorrhizall species. Continuous post-fire work should be undertaken in order to evaluate the long-term fire effects, on both flora and mycobiota diversity.

## Supplementary Material

064F11EB-0164-5CD5-AF32-991E57A50E59 10.3897/BDJ.7.e38177.suppl1 Supplementary material 1 Ascomycota and Basidiomycota macrofungi recorded in Mata da Margaraça Data type: Ecology, Occurrences, Trophic group, Taxonomy
Brief description: Species are arranged alphabetically according to higher taxonomic placement (Filo, Order and Family). Trophic group; P: parasitic; S: saprophytic; M: mycorrhizal. Host species (putative host); C: Castanea sativa; E: Eucalypthus spp.; H: Stereum spp.; I: Ilex aquifolium P: Pinus pinaster; Q: Quercus robur; T: Thaumetophoea pityocampa; Z: Peniophora spp. Novelties; N: novelties to Portugal; n: novelties to Beira Litoral. Occurrence; 1: recorded only in one of the two studies; 2: recorded in the two studies.
File: oo_329026.pdf https://binary.pensoft.net/file/329026 Natario B., Louro R. and Santos-Silva C.

## Hosting institution

Évora University
