# Supplementary material for: Macrofungi of Mata da Margaraça (Portugal), a relic from the Tertiary Age
Source: Biodivers Data J. 2019 Oct 3;7:e38177. doi: 10.3897/BDJ.7.e38177 (PMC6787105; doi:10.3897/BDJ.7.e38177)
Supplement: Supplementary material 1 — Ascomycota and Basidiomycota macrofungi recorded in Mata da Margaraça [file bdj-07-e38177-s001.pdf]

Biodiversity Data Journal

Macrofungi of Mata da Margaraça (Portugal), A Relic from the Tertiary Age

Bruno Natário1, Rogério Louro1, Celeste Santos-Silva1
1Biology Department, Macromycology Laboratory, Instituto de Ciências Agrárias e Ambientais Mediterrânicas, University of Évora, 7002-554 ÉVORA, Portugal

Table 1. *Ascomycota* and *Basidiomycota* macrofungi recorded in Mata da Margaraça. Species are arranged alphabetically according to higher taxonomic placement (Filo, Order and Family). Trophic group; P: parasitic; S: saprophytic; M: mycorrhizal. Host species (putative host); C: *Castanea sativa* ; E: *Eucalypthus* spp.; H: *Stereum* spp.; I: *Ilex aquifolium* P: *Pinus pinaster* ; Q: *Quercus robur* ; T: *Thaumetophoea pityocampa* ; Z: *Peniophora* spp. UEVH_FUNGI; Herbarium accession number or p.c.: private collection . Novelties; N: novelties to Portugal; n: novelties to Beira Litoral. Occurrence; 1: recorded only in one of the two studies; 2: recorded in the two studies.

Filo	Order	Family	Species	Trophic group	Host species	UEVH- FUNGI	Novelties	Post-fire spp.	Occurrence
Ascomycota	*Incertae sedis*	*Incerta sedis*	*Thyronectria aquifolii* (Fr.) Jaklitsch & Voglmayr	P	I	2004687	N		1
	Geoglossales	*Geoglossaceae*	*Geoglossum umbratile* Sacc.	S		p.c.	N		1
	Helotiales	*Leotiaceae*	*Leotia lubrica* (Scop.) Pers.	S		p.c.	n		1
		Helotiaceae	*Bisporella citrina* (Batsch) Korf & S.E. Carp.	S		2004460	N		2
		Rutstroemiaceae	*Lanzia echinophila* (Bull.) Korf	S		p.c.	n		1
			Rutstroemia firma (Pers.) P. Karst.	S		2004527	n		2
		Sclerotiniaceae	*Moellerodiscus lentus* (Berk. & Broome) Dumont	S		2004148	N		1
	Hypocreales	*Cordycipitaceae*	*Cordyceps militaris* (L.) Fr.	P	T	2004214	n		1
	Leotiales	*Bulgariaceae*	*Bulgaria inquinans* (Pers.) Fr.	S		2004450	N		1
	Pezizales	*Ascobolaceae*	*Ascobolus carbonarius* P. Karst.	S		2004096	N	x	1
		Helvellaceae	*Helvella elastica* Bull.	M	C, P, Q	p.c.	n		1
			Helvella fibrosa (Wallr.) Korf	M	C, P, Q	2004127	N		1
			Helvella lacunosa Afzel.	M	C, P, Q	2004457	n		1
			Helvella leucomelaena (Pers.) Nannf.	M	P	2004699	n		1
			Helvella leucopus Pers.	M	C, Q	2004109	n		1
			Helvella macropus (Pers.) P. Karst.	M	C, P,Q	2004130	N		1
		Morchellacea	*Disciotis venosa* (Pers.) Arnould	S		2004046	N		1
			Morchella elata Fr.	S		2004153	n	x	1
			Morchella esculenta (L.) Pers.	S		2004206	n	x	2
			Morchella eximia Boud.	S		2004183	N	x	1
			Verpa conica (O.F. Müll.) Sw.	S		2004689	N		1
		Pezizaceae	*Peziza arvernensis* Roze & Boud.	M	C, Q	2004218	n		1
			Peziza echinospora P. Karst.	M	C, Q	2004175	N	x	1
			Peziza granularis Donadini	M	C, Q	2004181	N		1
			Peziza lobulata (Velen.) Svrček	M	C, Q	2004140	N		1
			Peziza michelii (Boud.) Dennis	M	C, Q	2004219	N		1
			Peziza phyllogena Cooke	M	C, Q	2004227	n		1
			Peziza praetervisa Bres.	M	C, Q	2004176	n	x	1
			Peziza queletii Medardi, Lantieri & Cacialli	M	C, Q	2004128	N		1
			Peziza sepiatra Cooke	M	C, Q	2004143	n		1
			Plicaria endocarpoides (Berk.) Rifai	M	C, Q	2004180	N	x	1
		Pyronemataceae	*Anthracobia macrocystis* (Cooke) Boud.	S		2004241		x	1
			Cheilymenia granulata (Bull.) J. Moravec	S		2004746	N		1
			Geopora arenosa (Fuckel) S. Ahmad	M	C, P, Q	2004126	N		1
			Humaria hemisphaerica (F.H. Wigg.) Fuckel	M	C, Q	p.c.	n		1
			Lamprospora trachycarpa (Curr.) Seaver	S		2004137	N		1
			Otidea alutacea (Pers.) Massee	M	C, P, Q	p.c.	n		1
			Otidea bufonia (Pers.) Boud.	M	C, Q	2004463	n		1
			Pyronema omphalodes (Bull.) Fuckel	S		2004137	N	x	1
			Scutellinia scutellata (L.) Lambotte	S		p.c.	N		1
			Tarzetta catinus (Holmsk.) Korf & J.K. Rogers	M	C, Q	2004686	n		1
			Tarzetta cupularis (L.) Svrček	M	C, Q	2004149	n		2
			Trichophaea woolhopeia (Cooke & W. Phillips) Boud.	M	C, Q	2004744	n	X	1
		Rhizinaceae	*Rhizina undulata* Fr.	P	P	2004097		X	1
		Sarcoscyphaceae	*Sarcoscypha coccinea* (Gray) Boud.	S		2004447	n		2
	Xylariales	*Xylariaceae*	*Xylaria hypoxylon* (L.) Grev.	S		2004435	n		2
Basidiomycota	*Incertae sedis*	*Incerta sedis*	Cotylidia undulata (Fr.) P. Karst.	S	C, Q	p.c.	N		1
	Incertae sedis	*Incerta sedis*	*Loreleia marchantiae* (Singer & Clémençon) Redhead, Moncalvo, Vilgalys & Lutzoni	S		p.c.	N		1
	Agaricales	*Amanitaceae*	*Amanita citrina* Pers.	M	C, P, Q	2004345			2
			Amanita gemmata (Fr.) Bertill	M	C, P, Q	2004690			2
			Amanita mairei Foley	M	P	2000322	n		1
			Amanita muscaria (L.) Lam.	M	C, P, Q	2004343			2
			Amanita pantherina (DC.) Krombh.	M	C, P, Q	2004225			2
			Amanita phalloides (Vaill. ex Fr.) Link	M	C, P, Q	2004390			2
			Amanita rubescens Pers.	M	C, Q	2004215			2
			Amanita vaginata (Bull.) Lam.	M	C, P, Q	2004237			2
		Agaricaceae	*Agaricus amanitiformis* Wasser	S		p.c.	N		1
			Agaricus cappellianus Hlaváček	S		p.c.	N		1
			Bovista aestivalis (Bonord.) Demoulin	S		2004451	n		1
			Bovista cunninghamii Kreisel	S		2004402	n		1
			Bovista delicata Berk. & M.A. Curtis	S		2004309	n		1
			Bovista plumbea Pers.	S		2004208	n		1
			Bovista promontorii Kreisel	S		2004222	n		1
			Calvatia candida (Rostk.) Hollós	S		2004351	n		1
			Cyathus striatus (Huds.) Willd.	S		2004430	n		1
			Cystolepiota adulterina (F. H. Møller) Bon	S		p.c.			1
			Lepiota castanea Quél.	S		p.c.			1
			Lepiota clypeolaria (Bull.) P. Kumm.	S		p.c.	n		1
			Leucoagaricus crystallifer Vellinga	S		2004392	N		1
			Leucoagaricus serenus (Fr.) Bon & Boiffard	S		p.c.	n		1
			Lycoperdon excipuliforme (Scop.) Pers.	S		2004352	n		1
			Lycoperdon perlatum Pers.	S		p.c.	n		1
			Macrolepiota mastoidea (Fr.) Singer	S		2004332	n		2
			Macrolepiota procera (Scop.) Singer	S		2003044			2
			Macrolepiota venenata Bom	S		2004459	N		1
		Bolbitiaceae	*Conocybe vestita* (Fr.) Kühner	S		p.c.	N		1
		Clavariaceae	*Clavaria flavipes* Pers.	S		2004513	N		1
		Cortinareaceae	*Cortinarius acutus* (Pers.) Fr.	M	P	p.c.	N		1
			Cortinarius anomalus (Fr.) Fr.	M	C, Q	p.c.	n		1
			Cortinarius balteatocumatilis Rob. Henry ex P.D. Orton	M	C, P, Q	2004321	N		1
			Cortinarius caperatus (Pers.) Fr.	M	C, P, Q	p.c.	N		1
			Cortinarius flexipes (Pers.) Fr.	M	P	2004747	N		1
			Cortinarius rigens (Pers.) Fr.	M	C, P, Q	p.c.			1
			Cortinarius saniosus (Fr.) Fr.	M	C, Q	p.c.	n		1
			Cortinarius stillatitius Fr.	M	P	p.c.	N		1
			Cortinarius trivialis J.E. Lange	M	C, Q	2004472	n		2
			Cortinarius vernus H. Lindstr. & Melot	M	C, Q	p.c.	N		1
		Entolomataceae	*Clitopilus prunulus* (Scop.) P. Kumm.	S		2004400			2
			Entoloma hirtipes (Schumach.) M.M. Moser	S		2004092	n		2
			Entoloma papillatum (Bres.) Dennis	S		2004696	n		2
			Entoloma sericeum Quél.	S		p.c.	n		1
		Fistulinaceae	*Fistulina hepatica* (Schaeff.) With.	S		2004549	n		2
		Hydnangiaceae	*Laccaria amethystina* Cooke	M	C, P, Q	2004540	n		2
			Laccaria fraterna (Sacc.) Pegler	M	C, P, Q	2004138	n		1
			Laccaria laccata (Scop.) Cooke	M	C, P, Q	2004697			2
		Hygrophoraceae	*Cuphophyllus pratensis* (Fr.) Bon	S		p.c.			1
			Cuphophyllus virgineus (Wulfen) Kovalenko	S		p.c.	n		1
			Hygrocybe acutoconica (Clem.) Singer	S		2004691			1
			Hygrocybe conica (Schaeff.) P. Kumm.	S		p.c.			1
			Hygrocybe chlorophana (Fr.) Wünsche	S		p.c.			1
			Hygrocybe miniata (Fr.) P. Kumm.	S		2004521			1
			Hygrocybe russocoriacea (Berk. & T.K. Mill.) P.D. Orton & Watling	S		2004519	n		1
		Hymenogastraceae	*Deconica crobula* (Fr.) Romagn.	S		2004199			1
			Galerina clavata (Velen.) Kühner	S		2003983	N		1
			Galerina marginata (Batsch) Kühner	S		p.c.	N		1
			Gymnopilus odini (Fr.) Bon & P. Roux	S		2004291	N		1
			Gymnopilus penetrans (Fr.) Murrill	S		p.c.	n		1
			Hebeloma crustuliniforme (Bull.) Quél.	M	C, Q	2004692			1
			Hebeloma leucosarx P. D. Orton	M	C, Q	p.c.	N		1
			Hypholoma fasciculare (Huds.) P. Kumm.	S		2004467			2
		Inocybaceae	*Crepidotus applanatus* (Pers.) P. Kumm.	S		2004364	N		1
			Crepidotus autochthonus J.E. Lange	S		2004516	N		1
			Crepidotus mollis (Schaeff.) Staude	S		2004546			1
			Crepidotus variabilis (Pers.) P. Kumm.	S		p.c.			1
			Inocybe assimilata Britzelm.	M	C, P , Q	2004110	n		1
			Inocybe calida Velen.	M	C, Q	2004541	n		1
			Inocybe dulcamara (Pers.) P. Kumm.	M	P	2004226	n		1

			Inocybe geophylla (Bull.) P. Kumm.	M	C, Q	2003374			1
			Inocybe lacera (Fr.) P. Kumm.	M	C, P , Q	2004228			1
			Inocybe pallida Velen.	M	P	2004694	N		1
			Inocybe rimosa (Bull.) P. Kumm.	M	P	2004529			2
			Inocybe rufoalba Sacc.	M	C, P , Q	2004141	N		1
			Inocybe splendens R. Heim	M	C, Q	2004112	n		1
			Phaeomarasmius erinaceus (Fr.) Scherff. ex Romagn.	S		2004441			1
			Simocybe centunculus (Fr.) P. Karst.	S		2004561	N		1
		Lyophyllaceae	*Ossicaulis lignatilis* (Pers.) Redhead & Ginns	S		p.c.	N		1
			Tephrocybe anthracophila (Lasch) P.D. Orton	S		2004307	N	X	1
		Marasmiaceae	*Marasmius cohaerens* (Pers.) Cooke & Quél.	S		p.c.	n		1
			Marasmius epiphylloides (Rea) Sacc. & Trotte	S		p.c.	n		1
			Marasmius oreades (Bolton) Fr.	S		2003975			1
			Marasmius rotula (Scop.) Fr.	S		p.c.			1
			Marasmius torquescens Quél.	S		p.c.	n		1
		Mycenaceae	*Hemimycena lactea* (Pers.) Singer	S		2004426			1
			Mycena abramsii (Murrill) Murrill	S		2004436	n		2
			Mycena acicula (Schaeff.) P. Kumm.	S		p.c.			1
			Mycena aetites (Fr.) Quél.	S		2004305	n		1
			Mycena arcangeliana Bres.	S		p.c.			1
			Mycena cinerella (P. Karst.) P. Karst.	S		p.c.	n		1
			Mycena erubescens Höhn.	S		p.c.	n		1
			Mycena flavescens Velen.	S		2004542	n		1
			Mycena galericulata (Scop.)	S		p.c.			1
			Mycena galopus (Pers.) P. Kumm.	S		2004306			1
			Mycena inclinata (Fr.) Quél.	S		p.c.			1
			Mycena maculata P. Karst.	S		2004745	N		1
			Mycena pearsoniana Dennis ex Singer	S		p.c.	N		1
			Mycena pseudocorticola Kühner	S		2004489	N		1
			Mycena renati Quél.	S		p.c.	N		1
			Mycena rosea Gramberg	S		2003607			1
			Mycena seynii Quél.	S		2004356			2
			Mycena vitilis (Fr.) Quél.	S		2004453			1
			Mycena stipata Maas Geest. & Schwöbel	S		p.c.	N		1
			Roridomyces roridus (Fr.) Rexer	S		p.c.	N		1
			Xeromphalina campanella (Batsch) Kühner & Maire	S		2004198	N		1
		Omphalotaceae	*Gymnopus androsaceus* (L.) Della Maggiora & Trassin.	S		p.c.	n		1
			Gymnopus dryophilus (Bull.) Murrill	S		2004231			1
			Gymnopus erythropus (Pers.) Antonín, Halling & Noordel.	S		2004427			1
			Gymnopus foetidus (Sowerby) P. M. Kirk	S		p.c.	n		1
			Gymnopus fusipes (Bull.) Gray	S		p.c.	N		1
			Rhodocollybia butyracea (Bull.) Lennox	S		2003912			1
		Physalacriaceae	*Desarmillaria tabescens* (Scop.) R.A. Koch & Aime	P	C, Q	2004344	n		1
		Pluteaceae	*Pluteus cervinus* (Schaeff.) P. Kumm.	S		2004234			2
			Pluteus salicinus (Pers.) P. Kumm.	S		p.c.	n		1
			Volvopluteus gloiocephalus (DC.) Vizzini, Contu & Justo	S		2003990			1
		Psathyrellaceae	*Coprinellus angulatus* (Peck) Redhead, Vilgalys & Moncalvo	S		2004414	n	X	1
			Coprinellus domesticus (Bolton) Vilgalys, Hopple & Jacq. Johnson	S		2002732	n		1
			Coprinellus micaceus (Bull.) Vilgalys, Hopple & Jacq. Johnson	S		p.c.			1
			Coprinopsis lagopides (P. Karst.) Redhead, Vilgalys & Moncalv	S		2004517	n		2
			Psathyrella corrugis (Pers.) Konrad & Maubl.	S		2004394			1
			Psathyrella hirta Peck	S		2004173	n		1
			Psathyrella multipedata (Peck) A.H. Sm.	S		2004290	n		2
			Psathyrella murcida (Fr.) Kits van Wav.	S		2004401	N		1
			Psathyrella pennata (Fr.) A. Pearson & Dennis	S		2004469	n	X	1
			Psathyrella piluliformis (Bull.) P. D. Orton	S		p.c.	n		1
			Psathyrella spadiceogrisea (Schaeff.) Maire	S		2004293	n		1
		Schizophyllaceae	*Schizophyllum commune* Fr.	S		2004434			2
		Strophariaceae	*Agrocybe praecox* (Pers.) Fayod	S		2004695			1
			Cyclocybe aegerita (V. Brig.) Vizzini	S		p.c.			1
			Pholiota brunnescens A.H. Sm. & Hesler	S		2004333	N	X	1
			Pholiota carbonaria (Fr.) Singer	S		2004437		X	1
			Pholiota lenta (Pers.) Singer	S		2004367	N		1
		Tricholomataceae	*Arrhenia griseopallida* (Desm.) Watling	S		2000601	N		1
			Clitocybe costata Kühner & Romagn.	S		2004503	n		1
			Clitocybe gibba (Pers.) P. Kumm.	S		2004346			1
			Clitocybe odora (Bull.) P. Kumm.	S		p.c.			1
			Clitocybe subspadicea (J. E. Lange) Bon & Chevassut	S		p.c.	n		1
			Collybia tuberosa (Bull.) P. Kumm	S		2004303			1
			Lepista nuda (Bull.) Cook	S		p.c.			1
			Paralepista flaccida (Sowerby) Vizzini	S		2004439	n		1
			Sphagnurus paluster (Peck) Redhead & V. Hofst.	S		p.c.	N		1
			Tricholoma acerbum (Bull.) Quél.	M	C, Q	p.c.			1
			Tricholoma fulvum (DC.) Bigeard & H. Guill.	M	C, Q	p.c.	n		1
			Tricholoma sulphureum (Bull.) P. Kumm.	M	C, Q	p.c.			1
			Tricholomopsis rutilans (Schaeff.) Singer	S		2004375			2
		Tubariaceae	*Tubaria furfuracea* (Pers.) Gillet	S		2004295			1
			Tubaria romagnesiana Arnolds	S		2004433	n		1
		Typhulaceae	*Typhula quisquiliaris* (Fr.) Henn.	S		2004537	N		1
	Boletales	*Boletaceae*	*Boletus edulis* Bull.	M	C, P, Q	p.c.	n		1
			Boletus reticulatus Schaeff.	M	C, P, Q	p.c.	n		1
			Boletus subtomentosus L.	M	C, P, Q	p.c.	n		1
			Caloboletus calopus (Pers.) Vizzini	M	C, P, Q	p.c.	n		1
			Caloboletus radicans (Pers.) Vizzini	M	Q	p.c.	n		1
			Hortiboletus rubellus (Krombh.) Simonini, Vizzini & Gelardi	M	C, Q	2004384	n		1
			Leccinum molle (Bon) Bon	M	C, Q	p.c.	n		1
			Neoboletus erythropus (Pers.) C. Hahn	M	C, P, Q	p.c.	n		1
			Phylloporus rhodoxanthus (Schwein.) Bres.	M	C, Q	p.c.	n		1
			Xerocomellus chrysenteron (Bull.) Šutara	M	C, Q	2004514			2
			Xerocomellus porosporus (Imler ex Watling) Šutara	M	Q	p.c.	N		1
		Diplocystidiaceae	*Astraeus hygrometricus* (Pers.) Morgan	M	C, P, Q	2004357			2
		Gyroporaceae	*Gyroporus cyanescens* (Bull.) Quél.	M	C, Q	p.c.	n		1
		Hygrophoropsidaceae	*Hygrophoropsis aurantiaca* (Wulfen) Maire	S		2004557			2
		Paxillaceae	*Paxillus involutus* (Batsch) Fr.	M	C, P, Q	2004349			2
		Rhizopogonaceae	*Rhizopogon luteolus* Fr.	M	P	2004072	n		1
		Sclerodermataceae	*Pisolithus arhizus* (Scop.) Rauschert	M	C, E, Q	p.c.			1
			Scleroderma cepa Pers.	M	C, P, Q	p.c.			1
			Scleroderma citrinum Pers.	M	C, Q	2004524			2
		Suillaceae	*Suillus bovinus* (L.) Roussel	M	P	p.c.	n		1
	Cantharellales	*Cantharellaceae*	*Cantharellus pruinosus* Peck	M	C, Q	2004230	n		1
			Craterellus lutescens (Fr.) Fr.	M	C, Q	2004520			1
			Craterellus tubaeformis (Fr.) Quél.	M	C, Q	p.c.			1
			Pseudocraterellus subundulatus (Peck) D.A. Reid	S		2004535	N		1
		Clavulinaceae	*Clavulina coralloides* (L.) J. Schröt.	M	C, P, Q	p.c.	n		1
			Clavulina rugosa (Bull.) J. Schröt.	S		2004216	n		1
		Hydnaceae	*Hydnum repandum* L.	M	C, P, Q	p.c.	n		1
			Hydnum rufescens Pers.	M	C, Q	2004363	n		2
	Dacrymycetales	*Dacrymycetaceae*	*Calocera cornea* (Batsch) Fr.	S		p.c.	N		1
			Calocera viscosa (Pers.) Fr.	S		2004446	N		2
	Gomphalales	*Gomphaceae*	*Ramaria stricta* (Pers.) Quél.	M	C, P, Q	p.c.	n		1
	Hymenochaetales	*Hymenochaetaceae*	*Coltricia perennis* (L.) Murrill	M	C, P, Q	2004372			2
			Hymenochaetopsis tabacina (Sowerby) S.H. He & Jiao Yang	S		2004748			1
			Inonotus hispidus (Bull.) P. Karst.	S		2004445			1
			Phellinus pomaceus (Pers.) Maire	S		2004693			1
		Schizoporaceae	*Xylodon flaviporus* (Berk. & M.A. Curtis ex Cooke) Riebesehl & Langer	S		2004393	N		1
	Polyporales	*Fomitopsidaceae*	*Laetiporus sulphureus* (Bull.) Murrill	S		2004998			2
			Daedalea quercina (L.) Pers.	S		2004158			1
		Polyporaceae	*Cerrena unicolor* (Bull.) Murrill	S		2004391			1
			Faerberia carbonaria (Alb. & Schwein.) Pouzar	S		2004407	n	X	1
			Panus conchatus (Bull.) Fr.	S		2004461	N		1
			Trametes pubescens (Schumach.) Pilát	S		2004308	n		1
			Trametes versicolor (L.) Lloyd	S		2004449			1
	Russulales	*Russulaceae*	*Lactarius acerrimus* Britzelm.	M	C, Q	2004558			1
			Lactarius aurantiacus (Pers.) Gray	M	C, Q	2004523	n		1
			Lactarius chrysorrheus Fr.	M	C, Q	2003107			1
			Lactarius controversus Pers.	M	C, Q	2004382	n		1
			Lactarius lacunarum Romagn. ex Hora	M	C, Q	2003343	n		1

			Lactarius pergamenus (Sw.) Fr.	M	C, Q	p.c.	n		1
			Lactarius piperatus (L.) Pers.	M	C, Q	2004210			1
			Lactarius vellereus (Fr.) Fr.	M	C, P, Q	2004320	n		1
			Russula acrifolia Romagn.	M	C, P, Q	p.c.	N		1
			Russula amoena Quél.	M	C, Q	2004235	n		1
			Russula anthracina Romagn.	M	C, P, Q	p.c.	N		1
			Russula aurea Pers.	M	C, Q	p.c.	n		1
			Russula brunneoviolacea Crawshay	M	C, Q	p.c.	N		1
			Russula cyanoxantha (Schaeff.) Fr.	M	C, P, Q	p.c.	n		1
			Russula delica Fr.	M	C, P, Q	2004423			2
			Russula foetens Pers.	M	C, P, Q	2004289			1
			Russula fragilis Fr.	M	C, P, Q	2003331	n		1
			Russula graveolens Romell	M	C, Q	2004525	n		1
			Russula grata Britzelm.	M	C, P, Q	2004428	n		1
			Russula intermedia P. Karst.	M	C, Q	p.c.	N		1
			Russula ionochlora Romagn.	M	C, Q	p.c.	N		1
			Russula melitodes Romagn.	M	C, Q	2004239	N		1
			Russula odorata Romagn.	M	C, P, Q	2004223	n		1
			Russula parazurea Jul. Schäff.	M	C, Q	p.c.	n		1
			Russula pelargonia Niolle	M	C, Q	2004429	n		2
			Russula praetervisa Sarnari	M	C, P, Q	2004233	N		1
			Russula risigallina (Batsch) Sacc.	M	C, P, Q	2004688	n		1
			Russula virescens (Schaeff.) Fr.	M	C, Q	p.c.	n		1
		Stereaceae	*Aleurodiscus amorphus* (Pers.) J. Schröt.	S		2004312	n		1
			Stereum hirsutum (Willd.) Pers.	S		2004313			1
			Stereum subtomentosum Pouzar	S		2004311	n		1
	Tremellales	*Tremellaceae*	*Phaeotremella foliacea* (Pers.) Wedin, J.C. Zamora & Millanes	P	H	2004373	n		2
			Tremella mesenterica Retz.	P	Z	2004462	n		2
